# Dynamic Physical Distortions of Butterfly Pupal Wings: Potential Mechanical Signals from Eyespot Organizers for Color Pattern Determination

**DOI:** 10.3390/biology15110856

**Published:** 2026-05-29

**Authors:** Yugo Nakazato, Euichi Hirose, Joji M. Otaki

**Affiliations:** 1 The BCPH Unit of Molecular Physiology, Department of Chemistry, Biology, and Marine Science, University of the Ryukyus, Nishihara 903-0213, Okinawa, Japan; nakazato_s0mz@cs.u-ryukyu.ac.jp; 2 Tunicate Laboratory, Department of Chemistry, Biology, and Marine Science, University of the Ryukyus, Nishihara 903-0213, Okinawa, Japan; euichi@cs.u-ryukyu.ac.jp

**Keywords:** butterfly wing, color pattern formation, cuticle, eyespot, mechanical signal, morphogenic signal, organizer, pupal wing tissue, transmission electron microscopy

## Abstract

Butterfly wing color patterns are determined in pupal wing tissues, in which the prospective eyespot focus functions as a developmental organizer. Here, we investigated the microscopic structures of pupal wing tissues containing an eyespot organizer in line with the physical distortion hypothesis. Histochemical staining revealed that the pupal cuticle and epidermis were wavy and thin at 6 h but smooth and thick at 12 h postpupation. The eyespot organizer was associated with the thickest cuticle layer, called the cuticle focal spot. Transmission election microscopy (TEM) revealed that the intervening space (IVS) between the cuticle layer and the cellular apical end was wide at 6 h but narrow at 12 h postpupation. The spatial relationship between cuticle thickness and IVS width was indicative of mechanical buckling of the region adjacent to the cuticle focal spot. Live in vivo imaging revealed that the IVS at and near the eyespot organizer trapped orange fluorescent protein (OFP) injected into hemolymph. Dynamic distortions of the pupal wing epidermis and cuticular surface were detected in live individuals over time. These results suggest that physical distortions of the wing tissue induced by differential cuticle synthesis and subsequent buckling may function as mechanical morphogenic signals from eyespot organizers.

## Simple Summary

Butterfly wings often have eyespot color patterns, which are determined at the early pupal stage. Prospective eyespot focus in pupal wing tissues functions as a developmental organizer that releases morphogenic signals to surrounding cells. Here, we investigated the structures of pupal wing tissues containing an eyespot organizer at different time points after pupation using various microscopic techniques. We discovered several structural differences between time points and between organizing and nonorganizing cells. Cuticle thickness and the width of the intervening space (IVS) between the cuticle layer and the cellular apical end were indicative of mechanical buckling of the pupal wing tissue. The dynamic IVS, epidermal layer, and cuticular surfaces were detected by live imaging. These results suggest that physical distortions of the wing tissue induced by differential cuticle synthesis and subsequent buckling may function as mechanical morphogenic signals from eyespot organizers.

## 1. Introduction

The concept of organizers has been a central theme in embryology and developmental biology since 1924, when Spemann and Mangold demonstrated that dorsal blastopore lip tissue from the early gastrula of newts has an ability to induce neural fate in surrounding cells as a developmental organizer [1,2]. Since then, putative signaling molecules from organizers, now generally called morphogens, have been thought to be responsible for the induction process. This induction process is highly precise beyond individual differences in size and other variables, and it is highly robust against developmental and environmental noise. According to the gradient model for positional information proposed by Wolpert in 1969 [3], morphogens are defined as diffusible molecules that are produced and spread from source cells (i.e., an organizer) to form a stable concentration gradient, and morphogens are supposed to instruct undifferentiated cells to commit their defined fates in a direct concentration-dependent manner [4,5,6,7,8,9,10,11]. Developmental information is believed to be contained in molecular distributions. Representative morphogens that are known to be involved in the induction process are TGFβ family proteins, including BMP and activin [12,13,14,15,16,17,18,19,20], and Wnt family proteins [21,22]. However, how these morphogen molecules confer positional information to undifferentiated cells through actual spreading has been a matter of debate.

The prototype of the concentration gradient model requires the diffusion of morphogens across distances from an organizer and predetermined cellular thresholds in undifferentiated cells [3]. In this model, positional information is given to cells as a concentration cue. However, the concept of diffusible gradients was questioned by the original proposer [23,24] and other researchers [25,26,27]. This is mainly because simple diffusion does not appear to achieve stable gradients across variable distances within a limited period of time [23,24,25,26,27]. In other words, the flexible formation of diffusible morphogen gradients with respect to variable whole tissue size is not easily attainable, which is known as the scaling problem [28,29]. On the other hand, many studies have theoretically supported diffusion-based gradient models with some additional considerations. For example, the contribution of the extracellular matrix (ECM) may facilitate diffusion [30]. Heparan sulfate proteoglycans in the ECM may function together with Wnt and other morphogens to form and stabilize gradients [31,32,33,34]. Other proposed mechanisms for supporting gradients include, but are not limited to, mechanical cell competition [35,36,37], morphogen shuttling [38], extracellular vesicles [39], endocytosis [40], membrane association [41], and physiological modulation [42]. The scaling of morphogen distributions may be explained by the expansion–repression circuit implemented in morphogen gradient formation [43]. Reaction–diffusion systems may also be able to explain the scaling problem [44,45,46].

Experimental studies on diffusible morphogens have not kept up with the pace of these theoretical studies [47]. A recent experimental study revealed that the spread of the morphogen Decapentaplegic (Dpp) does not seem to be required for its function, which challenges the classical concept of morphogen spreading [48]. Similarly, Hedgehog morphogen gradients seem to form through molecular state transitions from membrane-confined and membrane-unconfined forms with diffusion barriers of cell–cell gaps, which is not explained by previous models [49]. Alternative mechanisms beyond diffusible morphogen gradients have also been investigated, including cell-to-cell signaling relay [50] and tissue growth [51]. Importantly, morphogens may be transported via cellular processes called cytonemes [52,53,54,55]. In this case, rather than undergoing extracellular spreading, morphogens may be directly and precisely transported via cytonemes, or actin-based cellular extensions, to undifferentiated cells to determine their fates [52,53,54,55].

These potential mechanisms of intercellular communication through molecular morphogens based on various types of delivery systems cannot exclude the possibility that other modes of signaling also occur during development. One of the important modes of developmental signals is physical (mechanical) signals. The idea of physical signals for embryonic development is not new; it may be considered as old as “developmental mechanics (Entwicklungsmechanik)” in the late nineteenth century proposed by Roux, Driesch, His, and others, who pioneered experimental embryology [56,57,58,59]. “Developmental mechanics” became obsolete in the twentieth century, but studies on mechanical signals in development were expanded and deepened by Gordon’s group in amphibian embryos [60,61,62,63,64,65,66]. It has been shown that the differentiation of epithelial cells is mechanically triggered by progressive differentiation waves via a cell state splitter located at the apical end of epithelial cells [60,61,62,63,64,65,66]. Recently, mechanical constraints from tissue interplay have been suggested to contribute to tissue morphogenesis in the field of developmental biomechanics [67].

**Figure 1:**
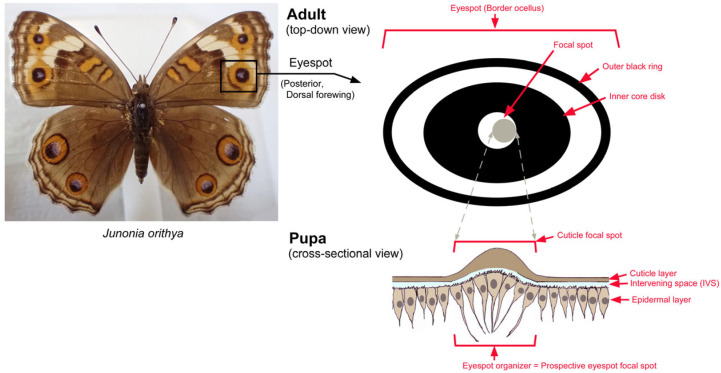
Correspondence among the focal spot at the center of the adult eyespot, the pupal cuticle focal spot, and the eyespot organizer (prospective eyespot focal spot; cells that provide surrounding undifferentiated cells with positional information for the eyespot). A dorsal view of a female individual of *Junonia orithya* (field-caught in May 1997 in Ishigaki-jima) is presented. The gray circle at the center of the adult eyespot in the illustration corresponds to the pupal cuticle focal spot.

Although amphibian embryos are often used for studying development, nymphalid butterfly wings are an excellent system for examining the functions of organizers and morphogens during development. Nymphalid butterflies such as those of the genus *Junonia* often have large eyespots on their wings, which are usually composed of a few rings around a white focal spot (Figure 1). Adult butterfly wings including their color patterns are determined in the early pupal stage from pupal wing tissues, in which epidermal (epithelial) cells form a single-cell epidermal sheet (Figure 1) [68,69,70]. Importantly, the prospective eyespot focal spot in the epidermal sheet functions as an eyespot organizer [70,71,72,73,74,75]. It is often presumed that the eyespot organizer releases diffusible morphogens for eyespot rings in accordance with concentration gradient models [70,71,72,73,76,77]. However, simple gradient models cannot explain the diversity of real (in contrast to idealized) butterfly eyespots nor several experimental results [74,78,79]. Importantly, the eyespot organizer is associated with the pupal cuticle focal spot on the surface of the pupal forewing, which is a local bump of the cuticle visually identifiable even by the human naked eye (Figure 1) [75,80,81].

Observational and experimental evidence suggests that this organizer-associated cuticle structure is not a nonfunctional entity but rather a functionally important structure; it may receive and release mechanical signals for eyespot formation from and to surrounding cells, which is known as the physical distortion hypothesis [82]. The functional importance of the pupal wing cuticle layer for wing color pattern determination is also referred to as the cuticle hypothesis [80]. These hypotheses are consistent with the original model for butterfly wing color pattern formation called the induction model, in which a black-inducing activator signal induces an inhibitory signal nearby [83]. These hypotheses and models are also consistent with the rolling ball model, in which morphogenic signals propagate in decelerating motion as if signals were composed of numerous rolling balls in accordance with Newtonian mechanics [84]. It should be stressed that these hypotheses and models have been proposed not on the basis of mathematical assumptions but on the experimental and observational results of pupal and adult wing structures and color patterns through inductive reasoning. Importantly, ectopic eyespots can be induced by physical damage and distortion [85], and eyespot formation requires physical contact of the pupal wing tissue with a reasonably hard surface [86,87], suggesting that morphogenic signals are generated by tissue distortion and travel with the help of mechanical force. An important finding for supporting these hypotheses is the discovery of wing membrane distortions at adult eyespot foci [88]. Additionally, several chemical compounds that can induce color pattern modifications such as tungstate and fluorescent brightener 28 (FB28) likely act on the extracellular chitin, one of the major components of the cuticle [89].

Notably, diverse butterfly color patterns are considered modifications of the general butterfly color pattern called the nymphalid groundplan, which was first proposed by Schwanwitsch [90] and Süffert [91] independently in the early twentieth century. The nymphalid groundplan was rediscovered by Nijhout [70,92] in the late twentieth century and was then elaborated by Otaki [93,94,95] in the early twenty-first century. The nymphalid groundplan states that the overall color pattern was constructed by color pattern elements that belong to symmetry systems. In addition to the nymphalid groundplan, several color pattern rules have been proposed [82]. One of them is the binary color rule. It states that the butterfly color patterns are primarily considered a mosaic of black and nonblack scales. It is black scales that constitute color pattern elements in the nymphalid groundplan, and nonblack colors are secondarily expressed, although the applicability of this rule to non-elemental patterns beyond the nymphalid groundplan has not yet been investigated. In other words, the binary rule states that undifferentiated scale cells have just two choices of developmental fates, i.e., to produce a black scale or to produce a nonblack scale. It is hypothesized that this binary fate choice may be achieved by the degree of cellular binding to the cuticle, which may be controlled by the mechanical buckling of the epidermis [96].

With these hypotheses and models in mind, here we examined the microstructures of pupal wing tissues using several microscopic techniques at two or three different time points during development. We examined prospective dorsal forewing eyespots, which are associated with cuticle focal spots [75,80,81]. The critical period for color pattern determination in this species was determined by cold shock treatment; severe modifications greatly decrease after 12 h of postpupation, although light modifications can be induced until 36 h of postpupation [97]. In a related species, the peacock pansy butterfly *Junonia almana*, a physical damage experiment revealed that organizing cells appear to function mainly before 18 h postpupation, and later damage likely induces injury effects that disrupt morphogenic signals [74]. That is, the function of organizing cells ceases approximately 18 h postpupation, although cellular competence for signal reception still remains to some extent until 42 h postpupation [74]. Accordingly, we primarily examined 6 h and 12 h postpupation tissues in the present study, which have not been observed before. The present results were also compared with the structures of pupal wing tissues within 1 h postpupation [80].

We paid particular attention to the extracellular space between the bottom of the surface cuticle layer and the apical end of epidermal cells, which is called the adhesion zone [98] or the intervening space (IVS). The IVS has not been examined in previous studies. We first observed fixed histological sections of pupal wing tissues with light microscopy for transmitted visible light, and we also observed fixed ultrathin sections using transmission electron microscopy (TEM). Moreover, we observed live organizers and their association with the cuticle layer in vivo using fluorescence confocal laser scanning microscopy (CLSM). We further observed the cuticular wing surface of live pupae using digital microscopy for reflected light. We discuss the potential mechanical signals that may operate in color pattern determination for butterfly wings.

## 2. Materials and Methods

### 2.1. Butterfly Samples

The present study focused on the blue pansy butterfly *Junonia orithya* (Linnaeus, 1758). We collected female butterflies on the Nishihara Campus of the University of the Ryukyus, Okinawa, Japan. Immediately after that, female butterflies were confined in a glass tank (300 mm × 300 mm × 300 mm) for oviposition in our laboratory. For egg collection, the natural host plant *Phyla nodiflora* was presented to the females. After hatching, the larvae were placed in a plastic container and given the natural host plant *Plantago asiatica* at approximately 27 °C under L16:D8 light conditions. This butterfly species is abundant throughout Okinawa-jima Island, Okinawa Prefecture, Japan, and no permission to collect and use it for research is necessary.

### 2.2. Microscopy

To prepare the pupal wing tissue sections for light microscopy and transmission electron microscopy (TEM), whole pupae were prefixed at 6 h and 12 h postpupation in a fixing solution of mildformR 20N (8% formaldehyde in phosphate buffer; pH 7.0–7.5) for weeks (FUJIFILM Wako Chemicals, Osaka, Japan) and stored at 4 °C. We first surgically dissected whole pupal wings and then excised the wing tissue regions containing the eyespot area. The isolated tissue samples were briefly rinsed in 0.1 M sodium cacodylate and postfixed in 1% osmium tetroxide in 0.1 M sodium cacodylate for 1.5 h at 4 °C. The samples were dehydrated through an ethanol series, rinsed with *n*-butyl glycidyl ether, and embedded in epoxy resin. The samples were cut to 0.5–1.0 μm thickness for light microscopy and approximately 0.1 μm thickness for TEM in the proximodistal direction. For light microscopy, tissue sections were stained with 1% toluidine blue, and the histological sections were examined at low magnification under a Nikon AZ100 multipurpose zoom microscope (Tokyo, Japan) with a Nikon digital sight DS-5Mc CCD camera. For TEM, ultrathin sections were stained with uranyl acetate and lead citrate. Stained sections were examined under a JEOL JEM-1011 TEM (Tokyo, Japan) at 80 kV. For histological and TEM observations, we used one pupal sample for each developmental time point.

For confocal laser scanning microscopy (CLSM), we used a Leica TCS SP8 DMI6000B confocal microscope system equipped with LASX (Leica Application Suite X) (Wetzlar, Germany). To do so, a pupal forewing was surgically lifted immediately after pupation (within 1 h) when the pupal cuticle was still soft. The exposed wing tissue was stained with BODIPY FL C_5_-ceramide complexed with BSA (henceforth called BODIPY) (Thermo Fisher Scientific, Tokyo, Japan) for membranous structures such as the plasma membrane via the sandwich method as described in previous studies [97,98,99,100]. The final concentration of BODIPY for the sandwich method was 16.9–25.3 µM. Orange/red fluorescent protein (henceforth called OFP; sold as OFPSpark) obtained from Sino Biological (Beijing, China) was simultaneously injected into the pupal abdomen. The dorsal hindwing surface was then placed on a thin glass plate for real-time in vivo observations. Due to the difficulty in observing the live dorsal forewing tissue in vivo, we observed the live dorsal hindwing posterior eyespot region with CLSM. The final concentration of OFP for injection was 1.85 nM. From the surface of the wing to deeper levels, horizontal optical serial sections were obtained to reconstitute vertical cross sections. For CLSM time-point observations, we used four pupal samples in total. At the time of these observations, the pupae were at 9–11 h or 3 h postpupation. For CLSM time-lapse observations, we used three pupal samples in total. In the first, second, and third samples, the observations were made in the period of 13–33 h, 12–32 h, and 5–35 h postpupation, respectively, with observational intervals of 20 min. Time-lapse movies were made at 3 fps to result in 1 h/sec (3600 times faster than the real-time movie).

To observe time-dependent changes in the dorsal forewings of live pupae, we used a Keyence Digital Microscope VHX-7000 (Osaka, Japan). No treatment of the samples was performed before the observations. To obtain cross-sectional height information for the surfaces, the brightness mode of this microscope was used. Pupae were examined at three time points, 6–8 h, 12–14 h, and 19–21 h postpupation. For the observations with the digital microscope, we used three pupal samples in total.

### 2.3. Image Analysis and Statistical Analysis

Acquired digital images were analyzed using ImageJ 1.54g (National Institute of Health, Bethesda, MD, USA) [101]. To obtain IVS area values and then IVS width without arbitrary measurements, TEM images (5000×) covering the IVS were vertically divided into 12 sections of equal width, and IVS areas were obtained in individual sections. Simultaneously, the lateral distances of the IVS were obtained. The average IVS width at each section was calculated on the assumption that the IVS area is rectangular in shape. The length of the short side of a theoretical rectangle with a measured area value was considered the IVS width. We measured the cuticle thickness and the IVS width at approximately the same sites after determining the correspondence between the light microscopy and TEM sections. Originally, candidate sites to be measured were determined to cover the entire histological sections with approximately equal intervals. However, due to the constraint that both histological and TEM sections should be measured at approximately the same sites, the final sites of measurements were limited to three and ten sites for the 6 h and 12 h sections, respectively. We treated these data as if they were randomly sampled. For statistical analyses, a two-sided unpaired Student’s *t* test was performed after the *F* test for equal variance. When necessary, due to unequal variance, Welch’s *t* test was performed. We used JSTAT 16.1 (Yokohama, Japan) and Microsoft Excel (Microsoft Office 365) for statistical analyses. Surface roughness was expressed as a ratio of curved surface length to the corresponding straight-line length.

## 3. Results

### 3.1. Histological Sections of Pupal Wing Tissues

**Figure 2:**
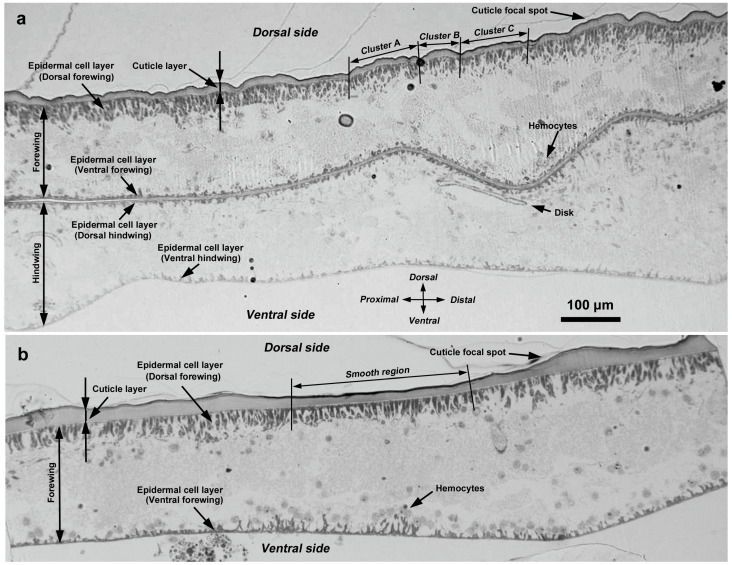
Histological images of pupal wing tissues including an eyespot organizer. An eyespot organizer is identifiable as epidermal cells below the cuticle focal spot. The scale bar in (**a**) is applicable to both panels. (**a**) At 6 h postpupation. A forewing and a hindwing are bound together but separated by cuticle layers in the middle of the tissue section. In addition to the cuticle focal spot, representative clusters (clusters A, B, and C) are indicated. A disk structure in the hindwing tissue is indicated, which may be a tracheole. (**b**) At 12 h postpupation. Only a forewing is observed in this tissue section. In addition to the cuticle focal spot, a smooth region is indicated.

To understand the overall structures of pupal wing tissues, we prepared histological tissue sections stained with toluidine blue at 6 h and 12 h postpupation, and they were observed under a bright light field (Figure 2). These 6 h and 12 h wing sections were similar in terms of overall structure; the surface of the tissue was covered with a cuticle layer, below which there was an epidermal layer. There was a relatively large hemolymph space between the facing dorsal and ventral epidermal layers. The dorsal epidermis was thicker than the ventral epidermis. Epidermal cells below the cuticle focal spot, i.e., organizing cells, appeared to be larger than the surrounding cells in both sections. Hemocytes were associated with epidermal cells.

More importantly, some traits differed between the 6 h and 12 h tissue sections. First, in the 6 h section, the dorsal surface of the cuticle layer was rough or wavy, on the basis of which the epidermal cells underneath seemed to form clusters (Figure 2a). In contrast, in the 12 h section, the dorsal surface of the cuticle layer was mostly smooth (Figure 2b). Most likely because of this smoothness, clusters were not clearly recognizable in the 12 h section. The cuticle layer in the dorsal forewing was thicker in the 12 h section (Figure 2b) than in the 6 h section (Figure 2a). Epidermal cells appeared to be slightly larger in the 12 h section (Figure 2b) than in the 6 h section (Figure 2a).

### 3.2. TEM Sections of Pupal Wing Tissues at 6 h Postpupation

**Figure 3:**
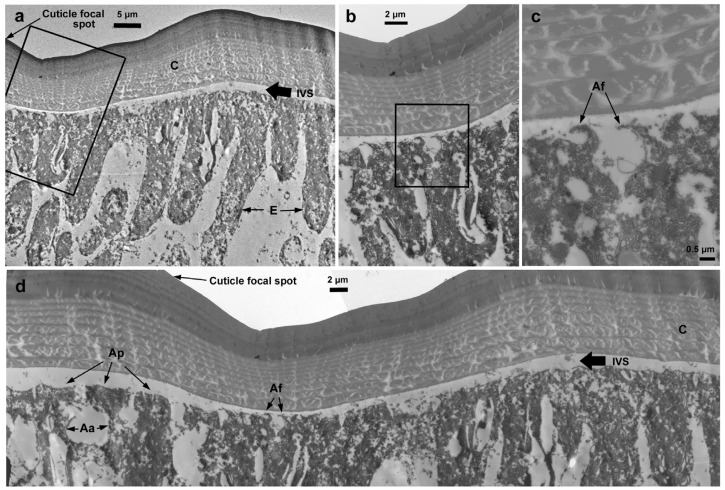
TEM images of pupal wing tissue at 6 h postpupation. C: cuticle, E: epidermal cell, IVS: intervening space, Aa: apical arm, Ap: apical palm, Af: apical finger. (**a**) Structures adjacent to the cuticle focal spot and eyespot organizer. A rectangular region is magnified in (**b**). (**b**) Enlargement of (**a**). A rectangular region is magnified in (**c**). (**c**) Enlargement of (**b**). (**d**) Wide apical view of (**a**).

We examined 6 h sections by TEM, paying attention to the IVS and the apical structures of the epidermal cells. Many epidermal cells were rod-like (columnar) in shape, and cells were connected laterally only at the apical end; the more basal side of the epidermal layer contained relatively large extracellular space (Figure 3a). The IVS was clearly identifiable between the inner surface of the cuticle layer and the apical surface of the epidermal cell layer. The apical end of the epidermal cells was not smooth but contained numerous microvillus-like fine structures; these structures were named apical fingers (Afs) (Figure 3b–d). The IVS immediately below the cuticle focal spot was relatively wide (Figure 3d). This was also true in the IVS below the cuticle bump just on the right of the cuticle focal spot (Figure 3d). The IVS between these two bumps was relatively narrow, as if this position of the cuticle were buckled down (Figure 3d). Nonetheless, the epidermal layer was curved mostly in parallel with the cuticle layer. Since the apical structures of epidermal cells were reminiscent of the structure of the human hand, we named the apical extension the apical arm (Aa) (Figure 3d). Similarly, the apical end of the apical arm was named the apical palm (Ap), from which Afs extended (Figure 3d). The Ap and Af may be analogous to the lamellipodium and filopodium, respectively.

**Figure A1:**
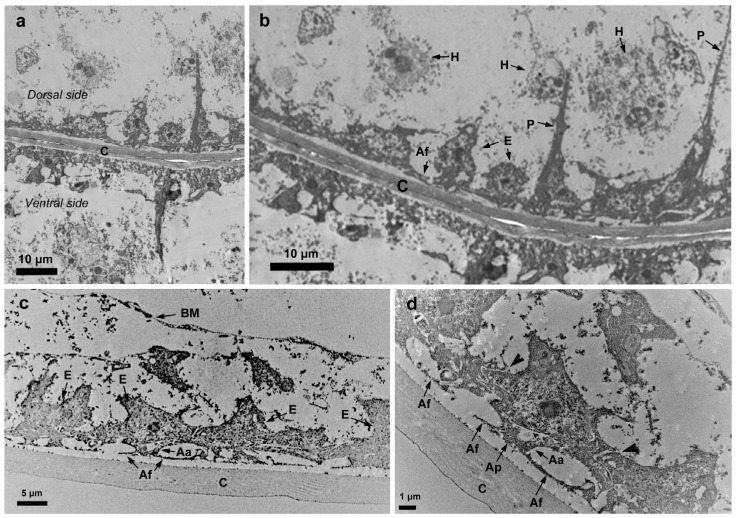
TEM images of pupal wing tissue (ventral forewing, dorsal hindwing, and ventral hindwing) at 6 h postpupation. (**a**) Boundary between the ventral forewing and the dorsal hindwing. The dorsal side is the forewing, and the ventral side is the hindwing. Two bound cuticle layers (C) are indicated. (**b**) Enlargement of (**a**). In addition to cuticle layers (C), hemocytes (H), epidermal cells (E), processes (P), and an apical finger (Af) are indicated. (**c**) Ventral epidermal cells in the hindwing. In addition to the cuticle layer (C), apical fingers (Af), an apical arm (Aa), epidermal cells (E), and basal membrane (BM) are indicated. (**d**) Enlargement of (**c**). Additionally, an apical palm (Ap) is indicated. Arrowheads indicate cellular gaps.

**Figure A2:**
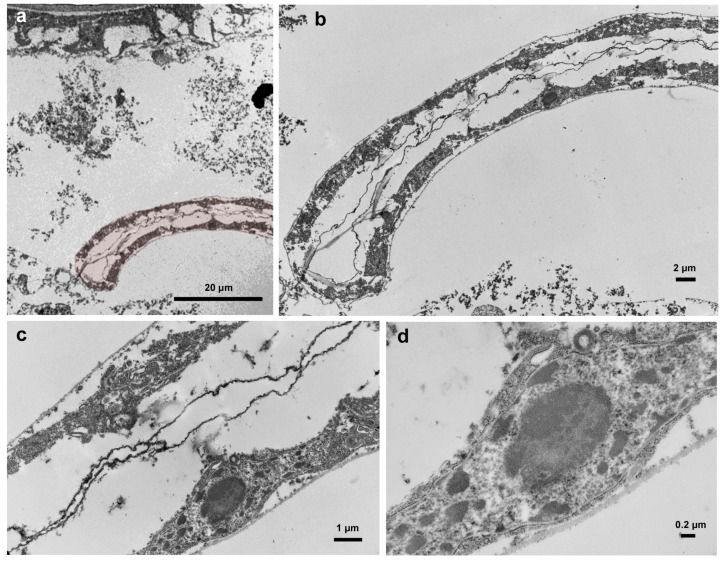
TEM images of pupal hindwing tissue with a disk structure 6 h postpupation. (**a**) Dorsal epidermis of the hindwing tissue with a disk structure (shown in pink). (**b**) Enlargement of (**a**). Cells of the disk structure are enclosed in a membrane. (**c**) Enlargement of (**b**). (**d**) Enlargement of (**c**).

We also observed epidermal layers of the ventral forewing, the dorsal hindwing, and the ventral hindwing (Appendix A.1 Figure A1). In the hindwing tissue, we observed the disk strucrure (Appendix A.2 Figure A2).

### 3.3. TEM Sections of Pupal Wing Tissues at 12 h Postpupation

**Figure 4:**
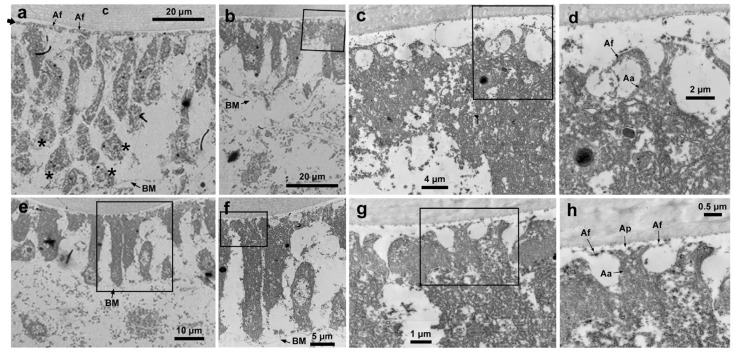
TEM images of pupal wing tissue at 12 h postpupation. The horizontal apical sheet (continuous Afs) placed closely to the cuticle layer is indicated by a bold arrow. C: cuticle, BM: basement membrane, Aa: apical arm, Ap: apical palm, Af: apical finger. (**a**) Structures at the cuticle focal spot and eyespot organizer. These Afs may be more like Ap extensions. Some cuboidal cells are indicated by asterisks. (**b**) Structures at the region that are different from (**a**). A rectangular region is magnified in (**c**). (**c**) Enlargement of (**b**). A rectangular region is magnified in (**d**). (**d**) Enlargement of (**c**). (**e**) Structures at the region that are further different from (**a**,**b**). A rectangular region is magnified in (**f**). (**f**) Enlargement of (**e**). A rectangular region is magnified in (**g**). (**g**) Enlargement of (**f**). A rectangular region is magnified in (**h**). (**h**) Enlargement of (**g**).

Here, we examined 12 h sections. Immediately below the cuticle focal spot, there were cells in which apical structures were elaborated to form a horizontal apical sheet placed closely to the cuticle layer (Figure 4a). The horizontal sheet appeared to be composed of Afs, but three-dimensionally, these Afs may be more like areal extensions of Aps, similar to lamellipodia instead of filopodia. In this cellular cluster, the epidermal cell bodies were irregularly arranged; there were cuboidal cells that may not have an apical extension (Figure 4a). This cellular configuration appears to be unique in this region at this time point and may be due to the globular clustering of organizing cells that underwent mitosis in the apicobasal direction. At the basal side, the basement membrane was observed, indicating the integrity of the epidermal layer. The basal processes of epidermal cells did not seem to break the basement membrane.

In a different but nearby region, there was a second type of cells in which apical structures were also elaborated with numerous Afs, but a clear horizontal sheet was not formed (Figure 4b–d). Similarly to the 6 h sections, the Aas and Aps were observed, and short Afs were in various directions, in which case small Aas were present in a self-similar fashion (Figure 4b–d). The basement membrane was also observed (Figure 4b). Additional sections showed cellular features in between; cells had multiple Aas, but Aps appeared to be aligned close to the cuticle layer (Figure 4e–h). Again, at the basal side, the epidermal layer was associated with the basement membrane (Figure 4e,f).

**Figure 5:**
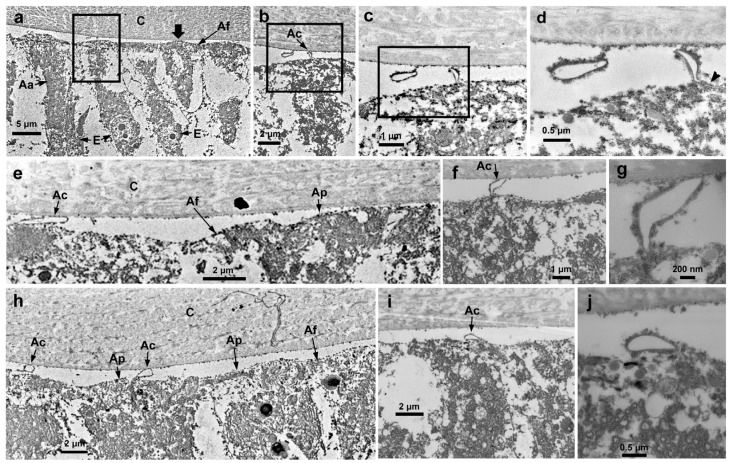
TEM images of pupal wing tissue at 12 h postpupation. C: cuticle, E: epidermal cell, Aa: apical arm, Ap: apical palm, Af: apical finger, Ac: apical cantilever. (**a**) Structures at the cuticle focal spot and eyespot organizer. A rectangular region is magnified in (**b**). A bold downward arrow indicates the direct contact point of a cell with the cuticle layer. Horizontally extending Afs are observed. These Afs may be more like Ap extensions. (**b**) Enlargement of (**a**). A single Ac is located at the center of a single Ap. A rectangular region is magnified in (**c**). (**c**) Enlargement of (**b**). A rectangular region is magnified in (**d**). (**d**) Enlargement of (**c**). Intracellular connection to the Ac is indicated by an arrowhead. (**e**) Structures at the cuticle spot and eyespot organizer. A different region from (**a**). (**f**) An Ac. (**g**) Enlargement of (**f**). (**h**) Structures at the cuticle spot and eyespot organizer. Another different region from (**a**) and (**e**). (**i**) An Ac. (**j**) Enlargement of (**i**).

In the other sections at the cuticle focal spot and eyespot organizer, we observed epidermal cells without elaborate Afs, and their apical ends were relatively flat (Figure 5a). Again, these flat Afs may be more like extending Aps, which may be analogous to lamellipodia rather than filopodia. Interestingly, however, there was a cantilever-like structure per cell at the center of a single Af, which was named the apical cantilever (Ac) (Figure 5b–j). The apical end of the Ac often contacted the inner surface of the cuticle. The Ac seemed to be directly connected to an intracellular structure (Figure 5d).

### 3.4. Cuticle Thickness and IVS Width

**Figure 6:**
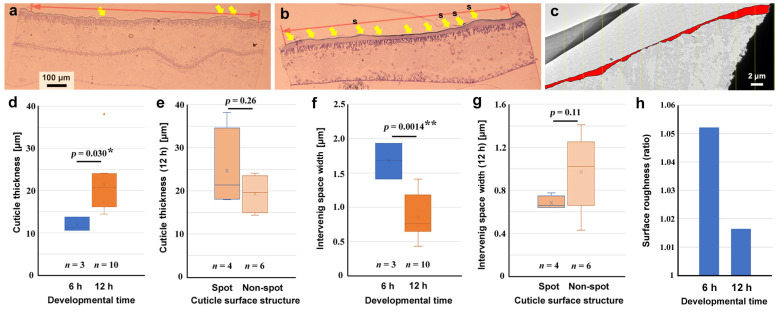
Quantitative comparisons between pupal wing tissues at 6 h and 12 h postpupation in terms of the cuticle layer thickness and the IVS width. Asterisks indicate statistical significance (two-sided unpaired Student’s *t* test; *: *p* < 0.05, **: *p* < 0.01). (**a**) Pupal wing tissue at 6 h postpupation stained with toluidine blue. This is the section identical to that in Figure 2a. The sites of measurements for cuticle width and IVS area are indicated by yellow arrows. The double-headed red arrow indicates the region for surface length measurement. The scale bar here is also applicable to (**b**). (**b**) Pupal wing tissue at 12 h postpupation stained with toluidine blue. This section is identical to that in Figure 2b. The sites for measurements of cuticle width and IVS area are indicated by yellow arrows. Among them, four spot regions are indicated by “s”. The double-headed red arrow indicates the region for surface length measurement. (**c**) An example of a TEM image for measurements of IVS area values shown in red. A single image was vertically divided into 12 sections of equal width. The area for each section was measured (see Section 2.3). (**d**) Box plots of cuticle width at 6 h and 12 h postpupation. (**e**) Box plots of cuticle width at the cuticle spot and at the noncuticle spot at 12 h postpupation. (**f**) Box plots of the IVS area at 6 h and 12 h postpupation. (**g**) Box plots of the IVS area at the cuticle spot and at the noncuticle spot at 12 h postpupation. (**h**) Surface roughness (ratio). The value 1 here indicates a straight line between the two endpoints of the surface length measurements.

Here, the cuticle thickness and the IVS width were quantified. They are likely two important factors for characterizing pupal wing tissues during development because the cuticle thickness could physically affect the facing IVS and epidermis. The IVS width may be considered an indicator of cell adhesion to the cuticle layer. We measured both the cuticle thickness and the IVS width at approximately the same sites after determining the correspondence between light microscopy sections and TEM sections (Figure 6a–c). The cuticle layer was significantly thicker in the 12 h section (21.50 ± 6.58 μm; henceforth the mean ± standard deviation) than in the 6 h section (11.82 ± 1.67 μm) (*p* = 0.030) (Figure 6d), indicating active secretion of cuticular components from epidermal cells during this period. In the 12 h section, the cuticle layer was thicker in the cuticle spots (including one middle spot and three points at and near the focal spot) (24.70 ± 9.38 μm) than in the noncuticle spots (19.37 ± 4.50 μm) (Figure 6b), but this difference was not statistically significant (*p* = 0.26) (Figure 6e). IVS width was significantly greater in the 6 h section (1.68 ± 0.26 μm) than in the 12 h section (0.86 ± 0.31 μm) (*p* = 0.0014) (Figure 6f), indicating that epidermal cells are more closely positioned to the cuticle layer and possibly have a higher level of cell adhesion at 12 h than at 6 h postpupation. In the 12 h section, the IVS width tended to be smaller in the cuticle spot area (0.68 ± 0.06 μm) than in the noncuticle spot area (0.97 ± 0.36 μm), but this difference was not statistically significant (*p* = 0.11) (Figure 6g). The cuticle surface was clearly rougher in the 6 h section (surface roughness ratio: 1.052) than in the 12 h section (surface roughness ratio: 1.016) (Figure 6h), indicating that there were more bumps in the 6 h section than in the 12 h section and suggesting that the cuticle surface was smoothened during this period, in accordance with qualitative observations.

**Figure 7:**
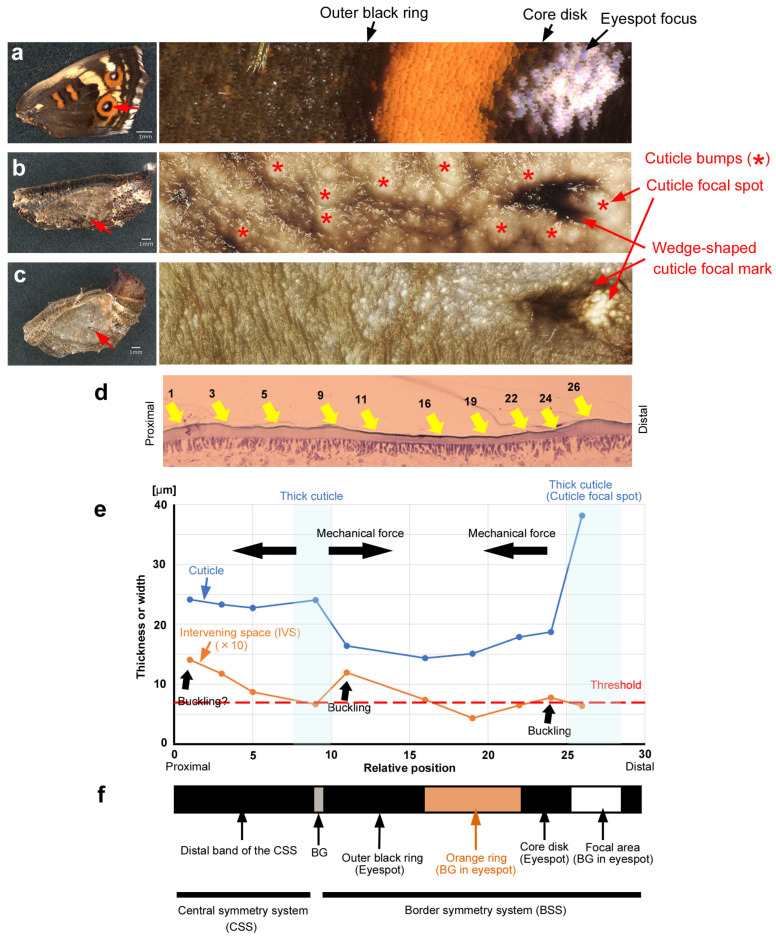
Correspondence among the adult eyespot, pupal cuticle focal spot, cuticle thickness, and IVS width. Panels (**a**–**f**) are arranged so that adult and pupal surface patterns can be visually compared. (**a**) Adult dorsal forewing color pattern with an eyespot immediately before eclosion. The posterior eyespot is indicated by a red arrow in the left panel, which is enlarged in the right panel. The eyespot focus, core disk, and outer black ring are indicated. (**b**) Pupal wing cuticle pattern at 12 h postpupation. The cuticle focal spot corresponding to the adult forewing posterior eyespot is indicated by a red arrow in the left panel, which is enlarged in the right panel. Some cuticle bumps are indicated by asterisks. The cuticle focal spot and wedge-shaped cuticle focal mark are indicated (also in (**c**)). (**c**) Pupal wing cuticle pattern after eclosion. The pupal case (exuvium) is shown. The cuticle focal spot corresponding to the adult forewing posterior eyespot is indicated by a red arrow in the left panel, which is enlarged in the right panel. (**d**) Sites of measurements for cuticle thickness (yellow arrows). The site numbers are indicated. IVS area values (the nearest ones to these sites) were also measured using TEM images (see Figure 6c). This image is identical to Figure 2b and Figure 6b. (**e**) Cuticle thickness and IVS width. Possible mechanical forces and buckling are indicated. The possible threshold line is indicated by a red broken line. (**f**) Adult wing color pattern generated according to the threshold set in (**e**). BG: background.

We further quantitatively examined the possible relationship between the cuticle thickness and the IVS width. To do so, we first compared adult and pupal structures (Figure 7a–c). In an adult wing before eclosion, scales were packed at high density, but an eyespot structure before the wing expansion was observed (Figure 7a). In a pupal wing, there were many cuticle bumps, and one of them was the cuticle focal spot, roughly corresponding to the adult eyespot focus (Figure 7b). There was a wedge-shaped cuticle mark at the proximal side of the cuticle focal spot (Figure 7b). The pupal case (exuvium) had a relatively smooth wing cuticle, but the cuticle focal spot and the cuticle focal mark were clearly observed (Figure 7c). Notably, the proximal end of the wedge-shaped cuticular mark corresponded to the proximal end of the eyespot black core disk. The correspondence of these adult and pupal structures with the 12 h section revealed that the smooth area (indicated in Figure 2b) roughly covered the eyespot area (Figure 7d). Moreover, the adult eyespot focus (corresponding to No. 26 in Figure 7d), the proximal end of the adult eyespot black core disk (corresponding to No. 22 in Figure 7d), and the end of the adult eyespot outer black ring (corresponding to No. 9 in Figure 7d) corresponded to the cuticle bumps on the surface of the cuticle layer (Figure 7d). When the cuticle thickness and the IVS area values were plotted together, these two plots were roughly mirror images as if the epidermal sheet were pushed up or down in response to the cuticle thickness (Figure 7e). Importantly, two sites (No. 11 and No. 24 in Figure 7d) immediately next to the thick cuticle sites showed relatively large IVS width values, which may be an indication of mechanical buckling of the wing tissue. A theoretical threshold value could be set to produce the adult wing color pattern based on the IVS width (Figure 7f). In this model, areas of large IVS width become black, and areas of small IVS width become nonblack, following a binary code (Figure 7f).

### 3.5. IVS in Live Wing Tissues

**Figure 8:**
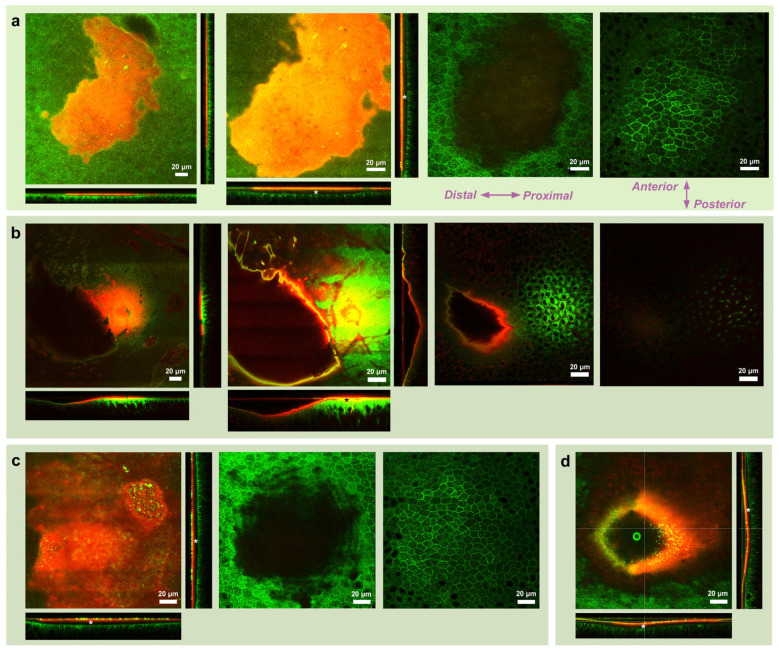
Live confocal images of the IVS corresponding to the posterior cuticle focal spot on the dorsal hindwing. Pupal wing tissue was stained with OFP (red) for the hemolymph space (IVS) and BODIPY (green) for membranous structures. Observations were made at 9–11 h postpupation except for the second individual at 3 h postpupation. IVSs are indicated by asterisks. (**a**) First individual. The leftmost panel is a low-magnification image with reconstituted vertical optical cross sections cut at the center of the image on the right and bottom. Horizontal optical sections were cut at 0.684 μm intervals. The depth values of the horizontal sections for these panels from left to right are 6.8 μm, 5.4 μm, 9.6 μm, and 14.4 μm from the top of the cuticle layer. Panels are arranged similarly in (**b**). The proximodistal and anteroposterior directions shown here are applicable to all panels in this figure. (**b**) Second individual. Horizontal optical sections were cut at 0.600 μm intervals. The depth values of the horizontal sections for these panels from left to right are 8.2 μm, 8.4 μm, 19.8 μm, and 33.6 μm from the top of the cuticle layer. (**c**) Third individual. Horizontal optical sections were cut at 0.502 μm intervals (also in (**d**)). The depth values of the horizontal sections for these panels from left to right are 6.0 μm, 10.0 μm, and 14.1 μm from the top of the cuticle layer. (**d**) Fourth individual. The depth value of the horizontal section for this panel was 7.5 μm from the top of the cuticle layer. The green circle at the center is an artifact.

Thus far, we have observed fixed tissue sections. To examine the presence of the dynamic IVS in vivo, we obtained live images of the cuticle focal spot in the dorsal hindwing using CLSM. To do so, OFP was injected into the abdomen of a pupa, and cells were stained with BODIPY. The IVS and epidermal cells corresponding to the cuticle focal spot were observed at 9–11 h postpupation except for the second individual at 3 h postpupation (*n* = 4) on the assumption that a region of large IVS width accumulates more OFP than other regions (Figure 8).

In the first individual, the IVS at the cuticle focal spot accumulated OFP (Figure 8a), and the IVS was larger at the center of the focal spot than that in adjacent regions. In the second individual, the IVS at the proximal region of the focal spot (corresponding to the cuticle focal mark) accumulated OFP, where the IVS was very narrow as shown by the yellow color (colocalization of OFP and BODIPY) (Figure 8b). In the third individual, the entire IVS region at the focal spot was stained red, but the IVS at the center of the focal spot was larger than that in adjacent regions (Figure 8c), as in the first case. In the fourth individual, the proximal region of the focal spot (corresponding to the cuticle focal mark) was mainly stained as in the second case, and this region had a relatively narrow IVS (Figure 8d). Throughout these four cases, it appeared that the IVS at the cuticle focal spot or its proximal region accumulated OFP. The staining variation among the four individuals probably reflects the dynamic nature of the IVS. Interestingly, in most cases, the inner surface of the cuticle layer instead of the entire IVS seemed to be stained intensively. This is probably because hemolymph may be retained more in the IVS either at the cuticle focal spot or at the proximal region. Additionally, new cuticle components may be actively added at the inner surface of the cuticle layer at these regions, and hemolymph proteins including OFP may have been passively incorporated into the cuticle layer.

### 3.6. Time-Dependent Changes in the Epidermal Layer

**Figure 9:**
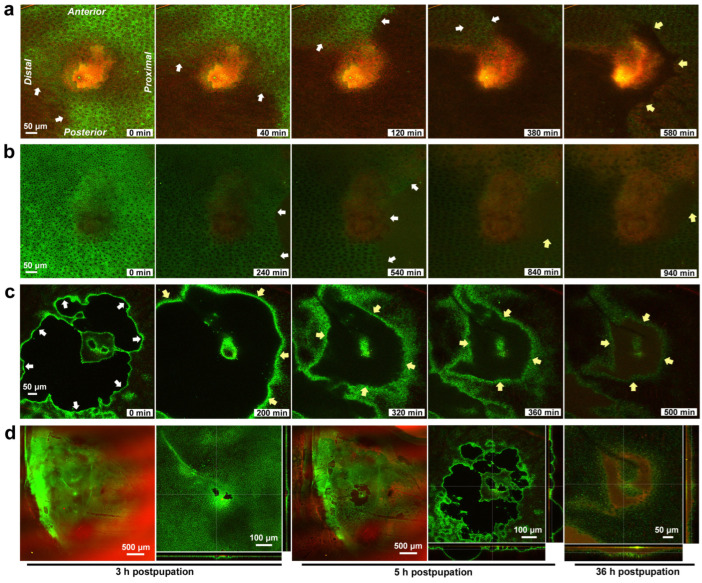
Live confocal time-lapse images of the epidermal layer corresponding to the posterior cuticle focal spot (organizing cells) on the dorsal hindwing. Pupal wing tissue was stained with OFP (red) for the hemolymph space (IVS) and BODIPY (green) for membranous structures. Observations were made for the 20 h or 30 h period with 20 min intervals. The expansion of the dark area (dent) was indicated by white arrows, and the expansion of the tissue area (recovery from dent) was indicated by yellow arrows. (**a**) First individual (13–33 h postpupation). The proximodistal and anteroposterior directions shown here are applicable to all panels in this figure. See Supplementary Video S1 for further details. (**b**) Second individual (12–32 h postpupation). See Supplementary Video S2 for further details. (**c**) Third individual (5–35 h postpupation). See Supplementary Video S3 for further details. (**d**) Additional images of the third individual before and after the time-lapse period. The left panels of the 3 h and 5 h postpupation images are low-magnification images. The right panels of the 3 h, 5 h, and 36 h postpupation images are high-magnification images with reconstituted vertical optical cross sections cut at the center of the image on the right and bottom.

The previous results suggest that the epidermal layer itself exhibits dynamic up-and-down movements that change the IVS width during development. To demonstrate this point, here we performed time-lapse imaging on the cuticle focal spot (organizing cells) in the dorsal hindwing using CLSM. As for the previous live imaging (Figure 8), OFP was injected into the abdomen of a pupa, and the epidermal cells around the cuticle focal spot were stained with BODIPY and observed during the 20 h or 30 h period with 20 min observational intervals (*n* = 3) (Figure 9).

In the first individual, the dark area (dent) of the epidermal layer expanded from the distal and posterior side toward the organizing center from 0 min (13 h postpupation) to 380 min (19.3 min postpupation), but the IVS at the organizing center did not change much (Figure 9a; Supplementary Video S1). Then, the dark area decreased from the proximal side (Figure 9a). In the second individual, the dark area expanded from the proximal side from 240 min (16 h postpupation) to 540 min (21 h postpupation) and then decreased at the proximal side (Figure 9b; Supplementary Video S2). These first and second individuals showed similar spatiotemporal dynamics. In the third individual, the dark area expanded from 3 h postpupation to 5 h postpupation (0 min) in all directions from the organizing center and then decreased in all directions to 500 min (13.3 h postpupation) (Figure 9c,d; Supplementary Video S3). Remarkably, the third individual showed the dark area around the organizing center, which is reminiscent of the future eyespot. The third individuals showed somewhat different dynamics from the first and second individuals, in that a circular dent around the organizing center was not clear in the first and second individuals. Nonetheless, in these three cases, the slow occurrence of a dent and its recovery around the organizing center in the time scale of hours was consistently observed, suggesting the mechanically flexible nature of the epidermal layer with the exception of organizing cells.

### 3.7. Time-Dependent Changes in Pupal Wing Surface Structures

**Figure 10:**
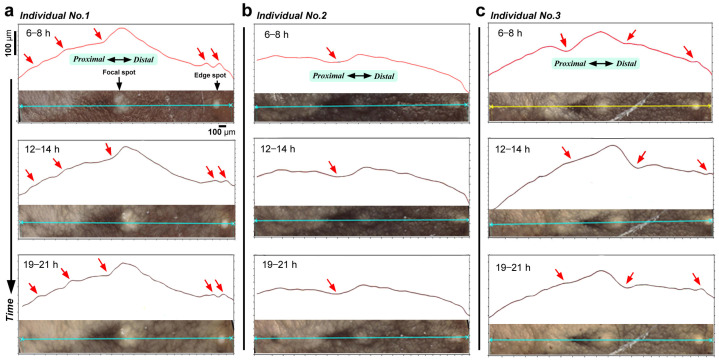
Time series of cross-sectional surface structures (covering the cuticle focal spot, the cuticle edge spot, and the cuticle middle spot) of the pupal wing. The results of three individuals are shown as height diagrams. The height diagram is combined with a cuticle surface image of the pupal wing, in which the measurement line is indicated by a double-headed blue–green arrow. Notable sites of height changes are indicated by red arrows. (**a**) First individual (individual No. 1). The pupal cuticle focal spot and edge spot are indicated. The scales here are applicable to all the panels in this figure. (**b**) Second individual (individual No. 2). (**c**) Third individual (individual No. 3).

Here, we examined whether live pupal surface structures change over time at three time points (6–8 h, 12–14 h, and 19–21 h postpupation) in a defined individual. A region of interest (ROI) was set to cover the cuticle focal spot, the cuticle edge spot (at the distal side from the focal spot), and the cuticle middle spot (at the proximal side from the focal spot) (*n* = 3) (Figure 10). In the first individual (individual No. 1), the shape and size of the focal spot, the edge spot, and their vicinity appeared to change over time (Figure 10a). New bumps also emerged on the proximal side at 19–21 h postpupation (Figure 10a). In the second individual (individual No. 2), the focal mark (located on the proximal side of the focal spot) appeared to have deepened slightly at 12–14 h postpupation, but the overall changes were not clear (Figure 10b). In the third individual (individual No. 3), the dent on the proximal side present at 6–8 h postpupation was smoothed at 12–14 h postpupation, and a new dent emerged on the distal side as if physical distortion waves of the cuticle were generated from or received at the cuticle focal spot (Figure 10c). Furthermore, the edge spot also showed a dynamic change over time (Figure 10c).

**Figure 11:**
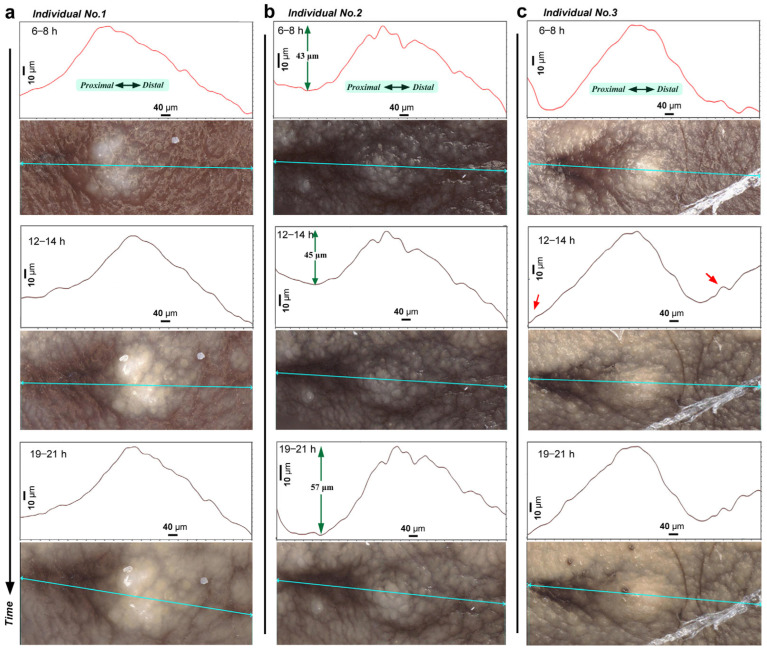
Time series of cross-sectional surface structures (near the cuticle focal spot) of pupal wings. The results of three individuals are shown as height diagrams (the same individuals shown in Figure 10). The height diagram is combined with a surface image of the pupal wing, in which the measurement line is indicated by a double-headed blue–green arrow. (**a**) First individual (individual No. 1). (**b**) Second individual (individual No. 2). The height of the cuticle focal spot is indicated. (**c**) Third individual (individual No. 3). Notable sites of height changes are indicated by red arrows.

To further investigate the surface dynamics, we obtained high-magnification images at three time points, focusing only on the focal spot in the same three individuals shown in the previous figure (*n* = 3) (Figure 11). In the first individual (individual No. 1), only small changes were observed around the focal spot (Figure 11a). In the second individual (individual No. 2), although no clear changes were observed at low magnification, the overall shape and size of the focal spot changed dramatically at high magnification, and the proximal side of the focal spot (the focal mark) deepened gradually (Figure 11b). In the third individual (individual No. 3), both the proximal and distal sides changed dramatically (Figure 11c). The proximal bump was smoothed as the focal mark elongated, and a new distal bump emerged (Figure 11c). In all three individuals, the shape of the focal spot, the focal mark, and other fine cuticular structures appeared to have changed over time in a waving movement.

## 4. Discussion

### 4.1. Dynamic Changes in Pupal Wing Tissues

In this study, we examined dynamic structural changes in butterfly pupal wings by comparing wing images at two main time points, at 6 h and 12 h postpupation, using TEM and other microscopic techniques, considering that the function of organizers seems to decline at 12–18 h postpupation [74,97]. Dynamic movements of live pupal wing tissues have been reported at the tissue level in other studies [100,102,103], and in those cases, tissue movements are associated with tissue size changes. In contrast, the present study examined time-dependent changes in cuticle surface structures, IVS, and epidermis at the histological, cellular, and subcellular levels in light of the physical distortion hypothesis for color pattern determination in butterfly wings.

Structurally, the 6 h tissue (Figure 2a) was largely similar to the 1 h tissue reported previously [80]. However, notable differences exist. The cuticle surface was smoother in the 1 h tissue than in the 6 h tissue. This finding is consistent with the hypothesis above because if the pupal surface structures mediate physical distortion waves for fate determination, we expect that such waves may be active during this period, i.e., during the functional period of organizing centers. Apical hand-like structures found in the 6 h and 12 h tissues (Aa, Ap, and Af) (Figure 3, Figure 4 and Figure 5) were not clearly observed in the 1 h tissue. This may simply be because the apical layer of the epidermal cells is more complex in the 1 h tissue, likely due to heavy cuticle secretion. Importantly, although there were numerous electron-dense dots and fibrous structures (likely the nascent cuticle) at the very apical end of the 1 h tissue [80], there were no such structures in the 6 h and 12 h tissues, suggesting that the highly active secretion of cuticle components decreased by 6 h postpupation. Consistent with this view, numerous vesicles with a core found in the 1 h tissue samples, which may be delivering the electron-dense dots (or similar ones) to the apical end [80], were not found in the 6 h tissue samples. Furthermore, numerous vacuole-like globules found in the 1 h tissue, which may contain ECM molecules on the basal side [80], were not present in the 6 h and 12 h tissues (Figure 3, Figure 4 and Figure 5), suggesting that active ECM secretion is over by 6 h postpupation. However, the results of the present study revealed that the cuticle layer thickness increased not only from 1 h to 6 h but also from 6 h to 12 h (Figure 6d), suggesting decreased but continuous secretion of cuticle components even after 6 h postpupation.

**Figure 12:**
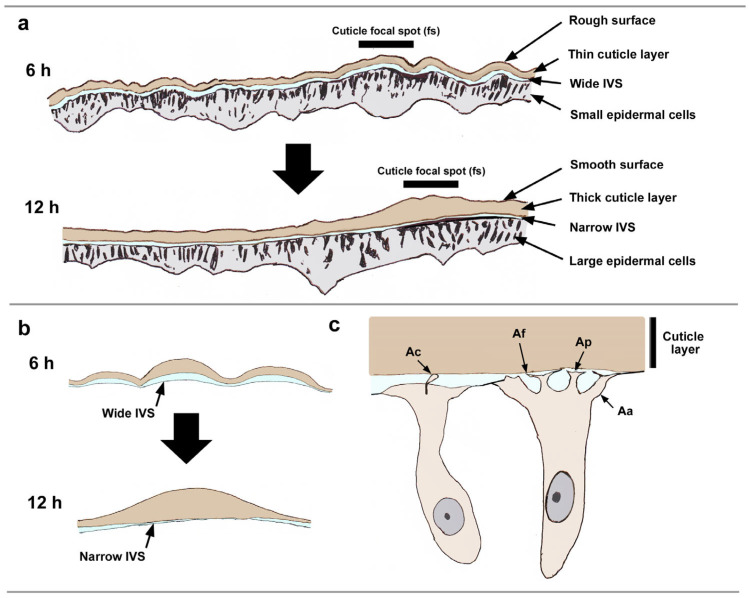
Summary of microscopic observations of histological and TEM sections at 6 h and 12 h postpupation. (**a**) Cuticle, IVS, and epidermal structures. The IVS in this illustration is not depicted in proportion to other parts. Four differential traits between the 6 h and 12 h sections are indicated. (**b**) Dynamics of the cuticle and IVS. (**c**) Representative cellular structures. Aa: apical arm, Ap: apical palm, Af; apical finger, Ac: apical cantilever (only in the 12 h sections).

The overall structures of the 6 h and 12 h samples were similar, but more importantly, some structural differences were notable: (1) relatively rough (6 h) versus smooth (12 h) surfaces, (2) relatively thin (6 h) versus thick (12 h) cuticle layers, (3) relatively wide (6 h) versus narrow (12 h) IVSs, and (4) relatively small (6 h) and large (12 h) epidermal cells (Figure 12a). Among these four traits, we were able to obtain statistically significant differences in the second and third traits (Figure 6d,f). Differences between spots versus non-spots in cuticle thickness and IVS width were not statistically significant (Figure 6e,g), but this may be because of the small sample size. Comparisons among three time points (6–8 h, 12–14 h, and 19–21 h postpupation) in the same individual pupa demonstrated that the cuticle surface bumps and dents changed over time (Figure 10 and Figure 11). Surface images also indicated that the wedge-shaped cuticle focal mark appears to change in size and shape (Figure 11). It is surprising that the pupal cuticle surface is flexibly mobile. These notable changes in cuticle surface structures, especially in the third individual (individual No. 3) examined in the present study, may be an indication of physical distortion waves generated from or received at the cuticle focal spot. Variation in dynamic surface movement among the three individuals examined may simply reflect the color pattern variation in adult wings. Although further studies are necessary to confirm this interpretation, these results suggest that the cuticle layer is flexible during the functional period of organizing centers. Further changes in the surface structures may be produced later, considering that the pupal case (exuvium) has a deep dent called the V-shaped canyon just next to the cuticle focal spot [80].

This flexible feature of the cuticle layer is not trivial because this feature opens the possibility that the cuticle layer may be an active and direct medium of morphogenic signals for color patterns. Moreover, cuticle flexibility should be compatible with cuticle hardness. Notably, for eyespot morphogenic signals to propagate, a hard surface material is required [86,87], and several color pattern modifiers (such as tungstate and FB28) and cold shock treatments appear to affect color patterns by delaying cuticle sclerotization [89]. However, it is still possible to argue that the surface changes that were observed in this study occur as a “side effect” of sclerotization and that they have nothing to do with color pattern determination, because we merely presented structural evidence but we did not provide any functional evidence for the physical distortion hypothesis in the present study.

The IVS width tended to be narrow at the adjacent region of the cuticle focal spot in the 6 h tissue but tended to be wide in the 12 h tissue (Figure 12b), suggesting dynamic changes in the IVS width over time. We also confirmed the live presence of the IVS using CLSM (Figure 8), demonstrating that the IVS is not an artifactual product, although the live images were obtained from the prospective dorsal hindwing eyespots. Live images from the 3 h and 9–11 h tissues showed that the center of the region corresponding to the cuticle focal spot had a wider IVS than the adjacent regions. This result is similar to that for the 6 h TEM sections but not to that for the 12 h TEM sections. This discrepancy may originate from the fact that the hindwing tissue is developmentally behind the forewing tissue. More studies are necessary to resolve this issue.

We further performed CLSM time-lapse imaging and demonstrated the flexible nature of the epidermal layer (Figure 9). It is noteworthy that the dent was formed around the organizing center, which is reminiscent of the future eyespot. Notably, the organizing center was not mobile, probably because it is bound relatively tightly to the cuticle focal spot. The dent around the organizing center then disappeared but not completely. These image data suggest that the epidermal layer is mechanically dynamic, and these changes may be explained if the organizing center and the cuticle focal spot function as a mechanical source and/or sink of the cuticle and epidermal layers. The epidermal up-and-down movements are probably correlated with similar movements driven by the covering cuticle layer. These changes occurred in the period of several hours, which may be considered a slow epidermal wave.

Importantly, the IVS width variation has been theoretically proposed to be an indicator of cellular adhesion (or here called attachment) to the cuticle layer [96]. Intriguingly, the black areas and nonblack areas in the adult butterfly wing can be assigned in accordance with the pupal IVS width (Figure 7e,f). In *Drosophila*, cell adhesion (including cell-to-cell adhesion and cell-to-ECM adhesion) is a critical parameter for wing development [104,105,106,107], and mechanical forces seem to be generated via cellular growth and adhesion in the *Drosophila* wing [108,109,110,111,112]. This is also likely true in lepidopteran insects [113,114,115]. However, in the original physical distortion hypothesis [82] and in the subsequent buckling model [96], the cuticle layer is considered fixed, and mechanical forces are supplied through cellular volume changes together with cellular adhesion to the cuticle layer. Accordingly, theories on butterfly wing color pattern development should be revised; in addition to cellular volume changes and adhesion, cuticle dynamics that could generate mechanical forces should be considered.

### 4.2. Apical Structures

Epidermal cells form an apical sheet [80,98,99,100], but we discovered via TEM that epidermal cells had unique structures associated with the facing cuticle layer, here termed Aa (apical arm), Ap (apical palm), and Af (apical finger), analogous to human hand structures (Figure 12c). These were found in both the 6 h and 12 h tissues but were less clear in the 1 h tissues, because of more complex Af or microvillus-like structures. The functions of the Aa, Ap, and Af are not known, but they appear to make close contact with the facing cuticle layer as if the cuticle were held by an open hand. We imagine that an Af is more like microvillus or filopodium than an Ap, and an Ap is often present at the vertical end of an Aa, but the distinction between an Ap and Af may be ambiguous in the two-dimensional TEM images. This is especially true in the organizing cells with a horizontally extending Ap or Af (Figure 4a and Figure 5a). These extending horizontal structures may be more like lamellipodia than filipodia. We speculate that some of these apical structures may harbor mechanosensory receptors such as PIEZO and serve as mechanical sensors for cells to detect physical distortions of the epidermal layer.

Moreover, we discovered the Ac (apical cantilever) (Figure 12c), which was seen only in the 12 h tissue sample. A single cell had only one Ac at the center of the single Aa and Ap, and this cell did not have complex apical structures. Because of these structural features and because the function of organizing cells gradually ceases at 12 h, we speculate that the Ac may be an early scale evagination. This means that epidermal cells with an Ac are scale cells, whereas other cells without an Ac are non-scale cells or scale cells before Ac production. In the early stage of pupal wing development, we previously discovered intricate chitin structures within a live epidermal cell using a different species, *Zizeeria maha*, via CLSM [116]. The Ac is likely difficult to clearly observe by CLSM, but we detected possible structures for scale shaft and base in developing epidermal cells [116]. Moreover, differential cuticle production on the surface of the wings has been suggested in *Z. maha* [116]. The inner contents of the Ac could be investigated by TEM-EDS (energy-dispersive X-ray spectroscopy), which may clarify the nature of the Ac, if relatively heavy atoms are specifically present.

It is interesting to note that precise apical structures in the 12 h sample varied depending on subtle positions in the tissue. The eyespot organizer at this time point appears to form cellular clusters containing both columnar and cuboidal cells, and columnar cells form horizontal Afs or Aps that tightly attach to the inner surface of the cuticle layer (Figure 4a). In a different but nearby region, the horizontal Afs or Aps are present, but cuboidal cells are not present, and a single Ac is present per columnar cell (Figure 5a). We could not precisely tell their positional relations, but these results suggest that the cellular attachment to the cuticle layer may be important to enable them to function as organizing cells; they may have to sense or withstand horizontal forces. These structural variations may also suggest earlier differentiation of organizing cells than other epidermal cells as suggested in previous studies [82,99].

### 4.3. Buckling Model for Color Pattern Determination

**Figure 13:**
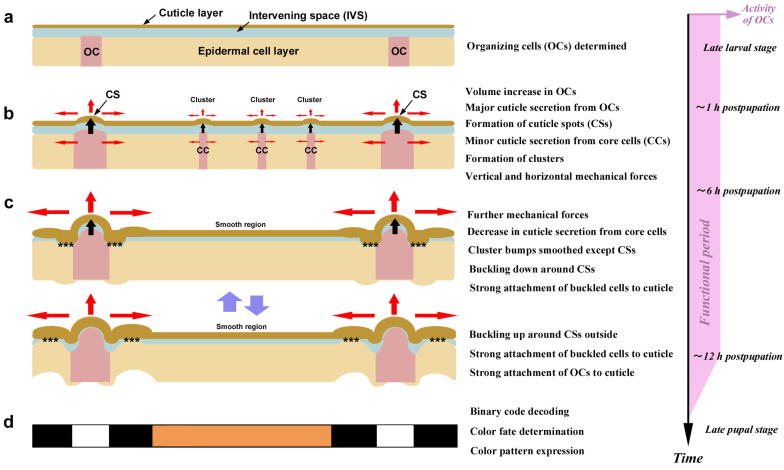
Buckling model for color pattern determination in butterfly wings. The functional period of organizing cells (OCs) is indicated on the right side of this figure. In this figure, physical contacts of the epidermal cell layer to the cuticle layer are not depicted, but these two layers are likely physically in contact via the apical structures of epidermal cells (i.e., Af, Ap, and Aa). (**a**) Locations of organizing cells (OCs) are determined during the late larval stage. At this point, it is assumed that the thin cuticle layer is evenly distributed because all epidermal cells are equally active in cuticle secretion. (**b**) Immediately before and after pupation, organizing cells secrete more cuticle components than their surrounding cells do, forming cuticle spots (CSs) and other small cuticle bumps. (**c**) After 6 h postpupation, excessive cuticle secretion produces vertical and horizontal mechanical forces. The horizontal forces from two organizing centers cause buckling of the cuticle around an organizing center. As a result, the epidermal cells in the buckled region make closer contact with the cuticle layer (asterisks). Buckling states may oscillate to generate ultraslow mechanical waves (top and bottom illustrations). Organizing cells also attach strongly to the cuticle. (**d**) The final color pattern is determined as binary code: a buckled region is expressed as black, and a nonbuckled region is expressed as a nonblack (orange in this case) color (background). The focal white is independent of this fate decision to some extent, probably according to the uncoupling rule.

There is the possibility that various characters of the cuticle layer (including cuticle thickness, IVS width, and surface roughness) might influence the developmental fate determination of epidermal cells. We have proposed a buckling model for color pattern formation, in which mechanical forces play a pivotal role [96]. In the previous model, the cuticle layer is considered fixed, and the epidermal layer accumulates forces from organizing centers due to an increase in organizing cells, either in volume or in number. In that case, the epidermal layer is buckled from the stable and hard cuticle layer in response to the horizontal forces. We indeed demonstrated the dynamic nature of the epidermal layer in time-lapse observations in the present study. Although this dynamic nature of the epidermal layer may be caused by forces from cellular changes in volume or in number, the present study additionally pointed out that the dynamic cuticle layer may cause changes in the IVS and epidermis. Taking the present results into account, here we propose a revised buckling model (Figure 13). It should be noted that the revised model is still hypothetical because we did not provide any direct evidence for functional signaling forces in this study.

To begin with, it is to be noted that the butterfly wing developmental system can be simplified as a system in which there are two flexible sheets (the cuticle and epidermal layers) stacked and lightly bound together. In such a tribological system, deformation of the top sheet directly deforms the bottom sheet. Here, we assume that the cuticle is an active changer as the top sheet, and the epidermis is a passive changer as the bottom sheet. We also assume that all epidermal cells below the cuticle layer are in contact with the cuticle layer. That is, the epidermal binding to the cuticle layer is assumed to be present at the molecular level, even though it is not visible in our TEM images. However, the binding level could be inferred from the IVS width.

First, the organizing centers (two organizing centers in this figure) are determined in wing tissue (Figure 13a). This determination is probably executed by the previous organizing centers in a self-similar fashion [95], but in this paper, this mechanism is not discussed. Organizing cells increase in volume, exercising lateral pressure on surrounding epidermal cells (Figure 13b). Simultaneously and more importantly, cuticle components are actively secreted from organizing cells. The secretion level is greater in organizing cells than in nonorganizing cells, resulting in the formation of a cuticle spot in the cuticle layer and the exercise of vertical and lateral pressures on the cuticle layer from the site of the cuticle spot. Cuticle components are also actively secreted from other cells, here termed core cells, which reside at the core of each cluster. However, the secretion level of core cells is much lower than that of organizing cells, and the activity of core cells produces small cuticle bumps on the surface of the cuticle layer. Secretion from core cells then ceases quickly, forming a smooth region (Figure 13c, top). The horizontal mechanical forces produced in the cuticle layer may result in the wavy surface found in the 6 h tissue sample. Importantly, mechanical stress in the cuticle layer also causes mechanical stress in the epidermal layer due to the binding of the epidermis to the cuticle layer.

Meantime, organizing cells tightly attach to the inner surface of the cuticle layer to release or withstand horizontal forces (Figure 13c, bottom). As the lateral mechanical forces in the cuticle layer increase, a region adjacent to the cuticle spot buckles down (inward buckling) (Figure 13c, top). The buckling direction is always inward first, at least in the dorsal forewing, because of the large cuticle spot above the cuticle plane. As a result, the epidermal cells below the buckled cuticle region are pushed down, and these cells and the cuticle layer make close contact with each other (shown by a relatively small IVS) at that time. This region may correspond to the focal mark located proximally from the focal spot. Alternatively, both the cuticle and epidermal layers are buckled together, and they slide over each other, in which case the IVS width at the buckled site may increase. Further forces may cause buckling upwards (outward buckling) in the region adjacent to the previously buckled-down region (Figure 13c, bottom). The buckled-up region now makes close contact with epidermal cells (shown by a relatively small IVS), but the previous buckled-down region may now lose close contact (shown by a relatively large IVS). Strong “attachment” of cells to the cuticle layer caused by even one instance of cuticle buckling may trigger the black-scale fate, whereas no buckling experience (“detachment”) may trigger only the nonblack-scale fate as the binary color code (Figure 13d). In other words, buckling mechanical stress imposed on epidermal cells functions as a morphogenic signal for eyespot color pattern determination. The IVS width is considered an indicator or code of the buckling activity of the cuticle layer.

As seen in this model, organizing centers are clusters of cells that produce more cuticle components than their surrounding cells do. Because all epidermal cells are likely attached to the cuticle layer at the molecular level, lateral expansion of the cuticle layer by differential cuticle secretion may directly generate mechanical forces in the apical sheet of epidermal cells. Consistently, it is known that epidermal cells in the basal area tilt greatly with deformed nuclei [99]. Furthermore, it is known that the cuticle focal spot size is correlated with the adult eyespot size [81]. We imagine that a large or small number of organizing cells produce a large or small cuticle focal spot, resulting in a large or small buckling area for a large or small eyespot, respectively. We have observed epidermal cells that may be specialized for high levels of chitin secretion in *Zizeeria maha* [116]. They may be considered organizing cells sensu lato.

In the previous buckling model, an attachment/detachment threshold is considered to determine the scale color fate, i.e., becoming black or nonblack, in a binary fashion, following the binary color rule [96]. On the basis of the IVS width, it is possible to set an attachment/detachment threshold for nonblack and black scales, respectively, as shown in Figure 7, but this is an opposite interpretation from the previous model [96] (in the previous model, we speculated that the “attached” state is translated into the black scale and the “detached” state into the nonblack scale). The IVS width may simply serve as an indicator of the degree of buckling experienced by epidermal cells.

### 4.4. Implications of the Buckling Model in Morphogenesis

Given that mechanical force generation and subsequent buckling in the cuticle layer are used as physiological signals for color pattern fate determination, how mechanical signals are interpreted as positional information should be explained at the molecular level. We believe that calcium waves are triggered by buckling movements. Wing-wide, long-range, and slow spontaneous calcium waves have been observed in butterfly pupal wings [117], and calcium signaling genes are expressed at the prospective eyespot [118]. Calcium waves are likely evoked by mechanical stress via mechanosensory receptors PIEZO1 [119] and TRPA1 [120], which are likely located in the apical membrane of epidermal cells. The signal transduction pathways would finally then act on gene expression changes. It should be noted that ectopic eyespots emerge at damage sites after damage-evoked calcium waves [117,121]. Physical damage may mimic physiological buckling in pupal wing tissues.

In addition to the differential secretion of cuticle components by epidermal cells, differential hardening of the cuticle may also be important in transducing mechanical signals. Hydrogen peroxide released from NADPH oxidase may play such a role [122]. Hydrogen peroxide may also directly modulate mechanosensory receptors such as PIEZO1 and TRPA1 [122]. Regional differences in the level of hydrogen peroxide may control the final color patterns. Furthermore, biogenic amines such as dopamine may contribute to cuticle hardening because they function as cold shock hormones for color pattern modifications in response to cold shock treatment [97]. The importance of cuticle sclerotization in insect development has been noted in several studies [104,105,106,107].

Mechanical buckling evoked by differential ECM secretion by cells may be a general mechanism for biological morphogenesis. Differentiation waves in axolotl embryogenesis [60,61,62,63,64,65,66] are probably similar to the physical distortion waves for butterfly wing color patterns proposed in the present study. Morphogenetic furrows in *Drosophila* eyes may be a type of mechanical differentiation wave [123,124,125,126]. A similar phenomenon is the generation of the cephalic furrow in *Drosophila* embryogenesis. The cephalic furrow is epithelial folding that is mechanically and genetically controlled [127]. Mechanical adjustments at the tissue level ensure precise epithelial folding [128,129,130]. Logically, F-actin is involved in the formation of the cephalic furrow [131]. Possible involvement of the actin cytoskeleton has been proposed in the butterfly wing system [119]. Importantly, plant morphogenesis is governed by mechanical forces generated by cellular activities, including cuticle deposition [132,133,134,135,136,137]. In this sense, plant and insect morphogenesis may be mechanistically similar to each other. Beyond biological systems, insect morphogenesis is analogous to plate tectonics in the earth crest and the formation of pancake color patterns if the buckling model is correct [96].

### 4.5. Mechanical Versus Molecular Signals

Mechanical signals have advantages over molecular signals in that mechanical signals do not seem to have problems with scaling, velocity, range, or gradient. Mechanical forces can spread instantly over long distances, although the spread of buckling waves may be very slow. We think that Wnt, Dpp, and other molecular morphogens in butterfly wings [138,139,140] may be expressed in response to calcium signals evoked by the buckling mechanical signals. Importantly, a previous TEM study [80] revealed that there seems to be no physical space suitable for morphogen spreading and gradient formation within 1 h of postpupation. This is partly because the IVS is busy incorporating cuticle components into the new cuticle layer. The present results also support this view at 6 h and 12 h postpupation, although cuticle secretion seems to be less active at these time points than at 1 h postpupation. Furthermore, the IVS is filled with dynamic hemolymph, as demonstrated by pharmacological injection treatments for modifying color patterns [89] and by the injection of OFP and other fluorescent dyes for live staining in previous [98,99,100,141] and present studies. Hemolymph circulation in the IVS seems to be reasonably fast, considering the fact that wing tissues are stained with fluorescent dyes almost immediately (3–10 min) after injection into the abdomen in our routine protocol [117]. Moreover, epidermal cells have unique structures on the apical side, i.e., Aa, Ap, Af, and Ac. The apical cell membrane in addition to the IVS may be too dynamic and too complicated to form a morphogen gradient during this period.

The extracellular space on the basal side may be a candidate site for morphogen spread, but this space, too, may not be free from the hemolymph current [80]. On the other hand, we could not detect cytonemes or other related structures for the delivery of molecular morphogens in previous [80] and present TEM studies. Therefore, prompt spreading and stable gradient formation may not be realistic until at least 12 h postpupation. We cannot exclude the possibility that such space for molecular morphogen spreading may be produced in later stages, but in that case, time is limited for organizer activity and molecular morphogen spreading.

Rather, molecular morphogens may be upregulated after mechanical signals are received by mechanosensory receptors and calcium signals are elevated in epidermal cells. In this scenario, mechanical signals (physical distortion) run first (“D” for distortion), which are translated into calcium signals (“C” for calcium), which then change gene expression for molecular morphogens (“G” for gene). These events may be repeated during development, which is called the DCG cycle [82]. Molecular morphogens may aid cellular adhesion to the cuticle without extensive spreading to refine the positional information provided by mechanical signals. That is, molecular morphogens may function to enhance cellular binding to the cuticle in response to mechanical signals. This feature of molecular morphogens could be very important because mechanical signals themselves may not last long. Physical distortion waves could not stimulate cells for a long time due to the refractory state of mechanosensory receptors. In other words, molecular morphogens may function to retain the “memory” of mechanical signals. Consistent with this hypothetical function of molecular morphogens, they bind to heparan sulfate proteoglycans in the ECM [31,32,33,34].

In the present study, OFP seems to be incorporated quickly into the cuticle layer probably due to the ongoing active growth of the cuticle layer. If molecular morphogens are secreted into the IVS, morphogen molecules will be “immobilized” or “embedded” quickly, like OFP, in new cuticle components actively secreted by epidermal cells. Embedded proteins may still be accessible from outside due to low cuticle density. In this way, molecular morphogens may be able to function as the molecular memory of mechanical signals. Cooperation of heparan sulfate proteoglycans and molecular morphogens [31,32,33,34] may be in line with this view. Interestingly, sulfated polysaccharides [142] along with tungstate [143] and FB28 [89] are color pattern modifiers in butterfly wings.

## 5. Conclusions

The results of the present study microscopically demonstrated that surface structures, cuticle thickness, and IVS width are dynamic at the early pupal stage in butterfly wings. We also showed that the epidermal layer itself is dynamic. These findings indicate that the cuticle layer is actively constructed by epidermal cells during the functional period of organizing cells and further suggest that the differential secretion of cuticle components by epidermal cells in wing tissue may produce position-dependent mechanical forces in the cuticle layer. In other words, the present study suggests that the function of organizing cells is to secrete more cuticle components than other cells do and that the real identity of morphogenic signals in a classical sense is mechanical forces that induce buckling. The buckling states of cells (i.e., cellular detachment from or attachment to the facing cuticle) may then determine the binary state of scale fates (i.e., black or nonblack scales to be produced). In this way, mechanical signals can provide undifferentiated cells with positional information. In this sense, it is likely that the insect pupal cuticle exoskeleton serves as a signaling medium (or “template”) for adult morphogenesis.

Overall, the present results support the physical distortion hypothesis and the cuticle hypothesis for color pattern determination in butterfly wings. Because the present supporting evidence for the physical distortion hypothesis is all based on microscopic image analyses, and because the buckling model is still hypothetical, physiological experiments for or against the hypothesis are expected in the future. We expect that the importance of the ECM and cellular adhesion in developmental fate determination will be revealed in other biological systems, including other insects and vertebrates.

## Data Availability

The original contributions presented in this study are included in the article. Further inquiries can be directed to the corresponding author.

## Supplementary Materials

The following supporting information can be downloaded at: https://www.mdpi.com/article/10.3390/biology15110856/s1, Supplementary Video S1: Time-lapse observations of the dent and its recovery in the epidermal layer (13–33 h postpupation) (Figure 9a); Supplementary Video S2: Time-lapse observations of the dent and its recovery in the epidermal layer (12–32 h postpupation) (Figure 9b); Supplementary Video S3: Time-lapse observations of the dent and its recovery in the epidermal layer (5–35 h postpupation) (Figure 9c).

## Abbreviations

The following abbreviations are used in this manuscript:

Aa	Apical arm
Ac	Apical cantilever
Af	Apical finger
Ap	Apical palm
CLSM	Confocal laser scanning microscopy
ECM	Extracellular matrix
EDS	Energy-dispersive X-ray spectroscopy
FB28	Fluorescent brightener 28
IVS	Intervening space
*J.*	*Junonia*
OFP	Orange/red fluorescent protein
TEM	Transmission electron microscopy
TRP	Transient receptor potential
*Z.*	*Zizeeria*

## Appendix A

### Appendix A.1. TEM Images of the Boundary Between the Forewing and the Hindwing

In this study, we focused mainly on dorsal forewing tissue. However, we also obtained some images of the ventral forewing, the dorsal hindwing, and the ventral hindwing (Appendix A.1 Figure A1). The boundary between the forewing and hindwing tissues showed cuticle layers that were tightly bound to each other with epidermal cells, and epidermal cells had long processes associated with hemocytes (Appendix A.1 Figure A1a,b). The epidermal cells seemed to be bound together on the apical side, forming a cellular sheet similar to that in the dorsal forewing. This is also true in the ventral hindwing tissue (Appendix A.1 Figure A1c,d).

### Appendix A.2. TEM Images of the Disk Structure in the Hindwing

We discovered the disk structure in the hindwing tissue at 6 h postpupation in the histological section (Figure 2a) and in the TEM section (Appendix A.2 Figure A2). This disk structure is probably a tracheole, which was previously observed in the wing tissue within 1 h postpupation [80]. The disk structure was enclosed with a membrane similar to the basement membrane of the epidermis. Inside, flat cells were located.
